# The emerging links between immunosenescence in innate immune system and neurocryptococcosis

**DOI:** 10.3389/fimmu.2024.1410090

**Published:** 2024-08-20

**Authors:** Luca Soraci, Alessia Beccacece, Maria Princiotto, Edlin Villalta Savedra, Maria Elsa Gambuzza, M’Hammed Aguennouz, Andrea Corsonello, Filippo Luciani, Lucia Muglia, Elvira Filicetti, Giada Ida Greco, Mara Volpentesta, Leonardo Biscetti

**Affiliations:** ^1^ Unit of Geriatric Medicine, Italian National Research Center on Aging (IRCCS INRCA), Cosenza, Italy; ^2^ Centre for Biostatistics and Applied Geriatric Clinical Epidemiology, Italian National Research Center on Aging (IRCCS INRCA), Ancona, Italy; ^3^ Independent Researcher, Messina, Italy; ^4^ Independent Researcher, Cosenza, Italy; ^5^ Territorial Office of Messina, Ministry of Health, Messina, Italy; ^6^ Department of Clinical and Experimental Medicine, Unit of Neurology and Neuromuscular Diseases, University of Messina, Messina, Italy; ^7^ Department of Pharmacy, Health and Nutritional Sciences, School of Medicine and Digital Technologies, University of Calabria, Arcavacata di Rende, Italy; ^8^ Dipartimento di Prevenzione, ASP di Cosenza, Cosenza, Italy; ^9^ Centre for Biostatistics and Applied Geriatric Clinical Epidemiology, Italian National Research Center on Aging (IRCCS INRCA), Cosenza, Italy; ^10^ Section of Neurology, Italian National Research Center on Aging (IRCCS INRCA), Ancona, Italy

**Keywords:** aging, IFN-I dysregulation, cryptococcal meningitis, vomocytosis, inflammaging

## Abstract

Immunosenescence refers to the age-related progressive decline of immune function contributing to the increased susceptibility to infectious diseases in older people. Neurocryptococcosis, an infectious disease of central nervous system (CNS) caused by *Cryptococcus neoformans (C. Neoformans)* and *C. gattii*, has been observed with increased frequency in aged people, as result of the reactivation of a latent infection or community acquisition. These opportunistic microorganisms belonging to kingdom of fungi are capable of surviving and replicating within macrophages. Typically, cryptococcus is expelled by vomocytosis, a non-lytic expulsive mechanism also promoted by interferon (IFN)-I, or by cell lysis. However, whereas in a first phase cryptococcal vomocytosis leads to a latent asymptomatic infection confined to the lung, an enhancement in vomocytosis, promoted by IFN-I overproduction, can be deleterious, leading the fungus to reach the blood stream and invade the CNS. *Cryptococcus* may not be easy to diagnose in older individuals and, if not timely treated, could be potentially lethal. Therefore, this review aims to elucidate the putative causes of the increased incidence of cryptococcal CNS infection in older people discussing in depth the mechanisms of immunosenscence potentially able to predispose to neurocryptococcosis, laying the foundations for future research. A deepest understanding of this relationship could provide new ways to improve the prevention and recognition of neurocryptococcosis in aged frail people, in order to quickly manage pharmacological interventions and to adopt further preventive measures able to reduce the main risk factors.

## 1 Introduction

Aging is a gradual and irreversible physiological process characterized by declines in tissue and cell functions, thus significantly increasing the risks for chronic diseases and concomitant disabilities and comorbidities (1). Immune function also decreases with age, due to a condition known as immunosenescence, which affects both natural and acquired immunity and puts older adults at increased risk of developing infections with a more severe and protracted course (2, 3). With advancing age and especially beyond the sixth decade of life, the human immune system undergoes aging-related dysregulated processes, associated with persistent low-grade inflammation, termed “inflammaging”, that involves multiple immune and non-immune cell types (3–5).

This aging-associated basal inflammation is thought to be induced by several factors, including the reactivation of latent infections and the engagement of pathogen-associated molecular patterns (PAMPs) and/or endogenous damage-associated molecular patterns (DAMPs), together with a dysregulation of specific host pattern recognition receptors (PRRs), among which the Toll-like receptors (TLRs) play a major role (3).

The dysfunctional immune response associated with both immunosenescence and inflammaging, responsible for a dysregulation of cytokine production and a persistent low-grade immune activation, may worsen the tissue damage caused by recurrent infections often affecting older subjects (6). Moreover, immunosenescence, frailty and comorbidities are conditions frequently associated with increased susceptibility to opportunistic infections, including fungal microorganisms, which usually become virulent with immunocompromised and unhealthy individuals (7–14).

Among the opportunistic infections affecting the central nervous system (CNS), meningitis and meningoencephalitis caused by *Cryptococcus neoformans* and *C. gattii*, also indicated as neurocryptococcosis, have been observed with increased frequency in HIV-seronegative individuals > 65 years, as result of a reactivation of latent infection, or community acquisition (15).

As documented by serological and epidemiological studies, the natural exposure to *Cryptococcus* sp. is very common in humans, despite overt clinical manifestations of disease are rare (16).

Protective immunity against cryptococcal yeasts is dependent on recognition, control, and proper interaction by and with cells of the innate and acquired immune response. In immunocompetent humans, the yeast, entered by inhalation and reached the lung alveoli, may be either completely cleared from the respiratory tract or establish a latent asymptomatic infection in pulmonary granuloma or thoracic lymph nodes. Depending on the host immune status and fungal virulence, cryptococcal organisms may cause progressive granulomatous inflammation, or form parenchyma granulomatous masses, known as cryptococcomas, very common in the lungs and brain of immunocompetent individuals (17, 18).

As with other organ systems, the CNS vulnerability to infectious agents increases with aging, and circulating microorganisms can invade the CNS, both by crossing the blood-brain barrier (BBB), or through transneuronal routes, causing infections in the meningeal or parenchymal compartments (15).

From a general point of view, the cryptococcal disease is favored by the presence of qualitative and/or quantitative alterations of immune system (e.g. HIV disease-related or drug-induced immune dysfunctions), even if CNS infection by cryptococcus may sometimes occur also in immunocompetent individuals (19). Although often overlooked, neurocryptococcosis is extremely risky in older people, due to a hardly diagnosis and its rapidly fatal disease course without prompt treatment; moreover, the incidence of neurocryptococcosis is expected to increase with the growing trend of aged people. While progresses have been made in understanding this disease in people living with HIV, data on older populations are scarce. To the best of our knowledge, this is the first review to examine the relationship between the senescence of innate immune responses and the development of cryptococcal meningitis in the elderly.

The connections among aging, immunosenescence, and neurocryptococcosis may indeed contribute to explain the increased risk of neurocryptococcosis in aged people (15, 20–22). In light of this, the purpose of this review is to identify mechanisms of susceptibility to cryptococcal meningitis and meningoencephalitis, with emphasis on the potential role of immunosenescence in innate immune cells. A deepest understanding of this relationship could lay the foundation for future research and provide new ways to improve the prevention and recognition of neurocryptococcosis in aged frail people, in order to quickly manage pharmacological interventions and to adopt further preventive measures able to reduce the main risk factors.

## 2 Neurocryptococcosis and aging: epidemiological evidence

Cryptococcal organisms are facultative intracellular pathogens which may commonly cause an invasive mycosis in immunocompromised individuals. Among the more of 30 *Cryptococcus* species, *C. neoformans* and *C. gatti* are closely related strains that cause respiratory and neurological diseases in humans and animals (23, 24). *C. neoformans*, which has a ubiquitous worldwide distribution, represents a common cause of meningitis in immunocompromised hosts (23). In contrast*, C. gattii*, which is geographically restricted to tropical and subtropical regions and is found less frequently in temperate regions, causes disease in both immunocompetent and immunocompromised hosts (25).

The infection, initially occurring in the lungs, upon inhalation of infective particles from the environmental source, is characterized by a wide array of clinical presentations. The fungal organisms can avoid the mucociliary clearance, and directly reach the alveolar spaces, where they are phagocyted by the lung tissue-resident immune cells. In healthy immunocompetent hosts, these fungi generally can either be successfully cleared or establish long-term, latent infections (26). In most cases, despite the exposure to cryptococcal cells is common, the development of symptomatic disease is rare and usually requires immunosuppression (24, 27), since the innate immune system is generally sufficient to limit the cryptococcal infection. However, in some subjects and often months or years after the exposure, the inhaled fungus can escape  the host’s defense mechanisms and disseminate from the lungs to the blood and invade the CNS, inducing likely fatal meningoencephalitis (28).

While there are several studies on cryptococcosis in immunocompromised hosts as people living with HIV, there are little data available to understand the presentation and the management of the cryptococcal infection in HIV-negative people (29–31). Generally, among patients with cryptococcosis, HIV-negative people are older than people living with HIV (32, 33). For instance, in a recent study, the median age of cryptococcal disease is 40 years in HIV people, 53 in non HIV transplanted people, and 61 in non-HIV people without history of transplantation (32). Different studies reported an higher mortality rate on HIV negative cohort compared to HIV-infected group (32, 34–37). Specifically, in a study by George et al. focused on the different epidemiology and outcomes between HIV and non-HIV patients with cryptococcosis, the authors found a lower median age (43.8 vs 58 years, p<0.001) and a lower overall mortality rate (25 vs 33.2%, p <0.001) in HIV compared to non- HIV subjects. These data support the importance of improving both diagnosis of cryptococcosis and therapeutic approach in HIV-negative patients.

This is a very relevant issue, also considering that incidence of neurocryptococcosis is increasing in HIV-negative people and in particular in the older adults (38). A recent population-based retrospective study comparing people living with HIV and HIV-negative people affected by meningoencephalitis have shown that up to 66.7% of HIV-negative patients were older than 50 years old. Furthermore, age >50 years and multiple chronic conditions were associated with higher mortality (30, 39). Another population-based study reported an increased rate of cryptococcal meningitis in the age group of 60-69 years, with a peak between 70 and 79 years (40).

Epidemiological evidence suggests that older patients (≥ 65 years), and especially those with underlying medical conditions, are more vulnerable to neurocryptococcosis than adults aged < 65 years (15, 22, 34, 36) and statistical analyses highlighted that age>60 years is a predictor of mortality (34, 41–43). In addition to older age, also impaired consciousness, hemodialysis, and previous corticosteroid usage have been associated with poor prognosis in HIV-negative patients with cryptococcal meningitis and could predict the higher mortality observed in this population compared with HIV patients (44). Furthermore, the not always overt presentation of CNS infection commonly displayed by elderly individuals makes it difficult to obtain an early diagnosis and to allow a timely therapy, thus potentially affecting the prognosis in a negative way (8, 45, 46).

Recent systematic studies focused on the identification of the risk factors mainly related to *C. neoformans* and/or *C. gattii* infections (47). According to these investigations, in addition to HIV-infected patients, the main groups of HIV-negative subjects at risk of a cryptococcal colonization and/or infection were aged people affected by immune dysregulation, lung dysfunction, kidney disease, cirrhosis, arthritis, diabetes, tuberculosis, connective tissue disorders, and patients receiving immunosuppressive therapies (47, 48). Other less commonly reported comorbidities associated to cryptococcosis are autoimmune disorders and sarcoidosis (47, 49–51).

Therefore, older age and the presence of chronic diseases are consistently associated with an increased risk of neurocryptococcosis. Nevertheless, a comparative analysis of healthy older people versus frail older people is lacking and should be further investigated. Moreover, the condition of frailty is never taken into account in any study.

## 3 Neurocryptococcosis and mechanisms of immune escape: the role of innate immunity

It is hypothesized that humans encounter the organism early in life, as shown by the gradual increase of cryptococcal-specific antibody response detected in humans with age (52). As most immunocompetent humans are asymptomatic and resolve the infection, there are limited observations concerning the mechanisms leading to cryptococcal clearance. However, the asymptomatic cryptococcal antigenemia, detected in the serum of HIV-infected people without signs or symptoms of meningitis or sepsis, indicates a relevant incidence of disseminated cryptococcal infection, at least in these subjects (53–55).

**Figure 1:**
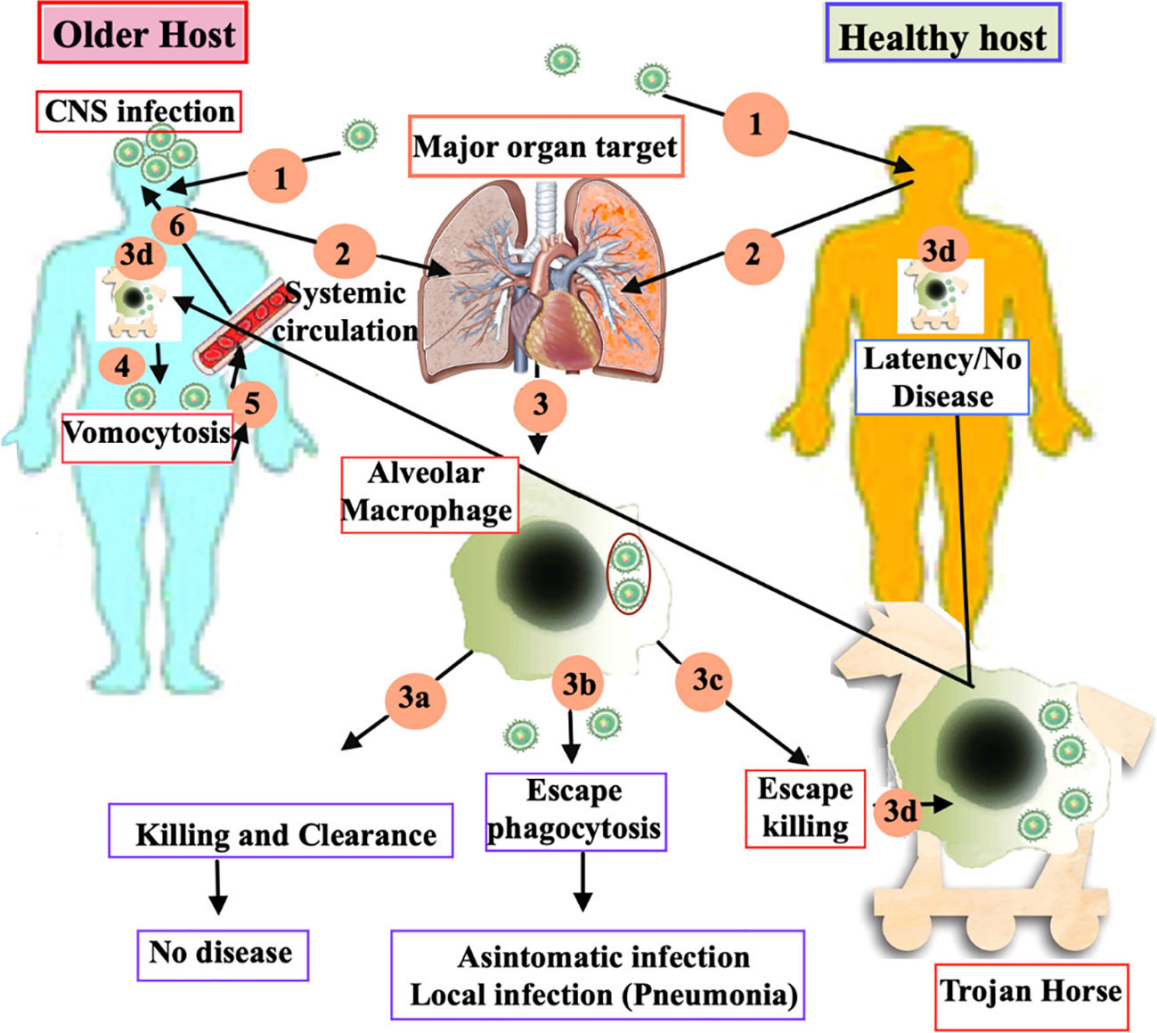
Current model of the immune response to Cryptococcus in older and young health host. **(1)** Primary cryptococcosis stars when dehydrated spores are inhaled into the lungs of both immunocompetent and immunocompromised older hosts. This is followed by the phagocytosis of the spores by the alveolar macrophages. **(2)**This activation triggers three different immune-responses of macrophage: **(3a)** successful killing and clearance, resulting in no disease; **(3b)** evasion of phagocytosis, leading to either an asymptomatic state or localized infection, such as pneumonia; and **(3c)** survival within the macrophages through evasion mechanisms, often likened to “Trojan horses” **(3d)**. During the latency stages, survival within macrophages and granuloma formation tends to be the predominant response. **(4)** In older hosts, reactivation occurs more frequently due to immune dysregulation. The spread of the pathogen beyond the respiratory system via vomocytosis leads to secondary cryptococcosis. **(5)** Cryptococcal cells, ejected through vomocytosis, enter the bloodstream and disseminate, resulting in a blood infection called cryptococcaemia. **(6)** After crossing the blood brain barrier, Cryptococcus migrates to the brain parenchyma and starts to proliferate, leading to fatal meningoencephalitis.

The natural history of human infection follows three steps: primary infection, followed by a silent phase of latency, that can last for years, and a last step consisting in the reactivation of dormant/latent fungal infection, associated with development of symptomatic disease, usually occurring in immune dysregulation conditions. Primary cryptococcosis, that initiates with lung involvement, can occur both in immunocompetent and immunocompromised hosts, as shown in **Figure 1**, and is triggered by inhalation of dehydrated spores, with subsequent phagocytosis of Cryptococcus by alveolar macrophages (56). The silent phase of latency can be characterized by the complete clearance of the fungus, or a latency of the disease, with the presence of the fungus in subpleural nodules and draining lymph nodes (57, 58). In any case, the survival inside macrophages and granuloma formation appears to be the predominant in latency stages (59). The reactivation, that may occur after the latency stage, is mainly dependent from two conditions: dormancy/survival of the fungi and immune dysregulation of the host, with subsequent development of secondary cryptococcosis.

*Cryptococci* can escape immune system mechanisms at several stages. One of the most important innate immune cells involved in fungal clearance are represented by dendritic cells and neutrophils; the binding of cryptococcal antigens to innate immune receptors expressed by lung dendritic cells induces the synthesis of specific cytokines able to stimulate macrophage polarization into M1 or M2 phenotypes, which guide the clinical outcome of lung infection (60); indeed, M1 cells are able to produce proinflammatory cytokines in large amount and are responsible of fungal clearance, in contrast to anti-inflammatory M2 cells which allow intracellular fungal replication; activated eosinophils contribute to macrophage polarization into M1 and cryptococcal clearance. However, multiple virulence factors, including polysaccharide capsule, melanin, and fungal proteins, allow the fungus to evade M1-mediated phagocytosis and further replicate within M2 cells (61).

Neutrophils are also important in the immune host response against the pathogen; Rocha et al. showed that neutrophils can form the neutrophil extracellular trap (NET) consisting of chromatin, cytosolic and granular proteins, in order to retain and kill the fungus (62); however, the glucuronoxylomannan capsule of *C. neoformans* may help the pathogen block the NET production and neutrophil migration. Indeed, in people with immune system dysfunctions, *Cryptococcus* may use escape mechanisms that favor its latent persistence in the lungs thanks to cell masking, N-glucan structures, and production of several enzymes and transcription factors (56).

In any case, prolonged persistence and replication of *Cryptococcus* within immune cells may further promote its dissemination outside of the respiratory system; indeed, the fungus uses phagocytes as reservoirs to replicate within and then be transported in the bloodstream, through a process of dissemination known as the “Trojan horse” (63). The phagocyted microorganisms, following intracellular replication, can be expelled by immune cells by both vomocytosis, that represents a mechanism of non-lytic exocytosis, and cell lysis (or rupture), due to excessive intracellular proliferation (27, 64). Vomocytosis can be also followed by phagocytosis by nearby immune cells, through a process considered a new escape mechanism and called dragotcytosis, characterized by the interaction between the donor and acceptor macrophages prior to and shortly after the pathogen transfer event (65).

Cryptococcal cells can also leave the lungs as free fungi via a number of extracellular routes, mainly including vesicular-mediated transcellular crossing, also known as transcytosis, and paracellular crossing, or paracytosis, which involves the mechanical or biochemical disruption of the tight junctions (63). Once outside of the lungs, cryptococcal cells enter the bloodstream and spread, leading to a blood infection known as cryptococcaemia, whose magnitude shows a direct correlation with severity of the infection (63). In any case, the fungi, following migration across the BBB via “Trojan horse” mechanism, transcytosis, paracytosis, and/or free entry through damaged endothelial barriers, migrate to the brain parenchyma and begin to proliferate, frequently causing fatal meningoencephalitis (66, 67).

Although *C. neoformans* and *C. gattii* enter the body through the lungs, both pathogens have indeed a strong affinity for the CNS (68). Since *C. neoformans* is able to survive within phagosome after being phagocytized by innate immune cells, the phagocytic cells play an essential role in the diffusion of the pathogen in the brain. Following CNS invasion, the fungal pathogens activate microglia, the brain-resident macrophages, which represent the largest population of myeloid cells in the CNS. Microglial activation induces the production of proinflammatory cytokines and chemokines, which in turn lead to neuroinflammation and promote the recruitment and accumulation of both innate and adaptive immune cells. The immune cells, along with the cytokines secreted by them, are critically involved in fighting the fungal cells.

A recent preclinical study highlighted the presence of monocytes, neutrophils, and proinflammatory cytokines in brain perivascular spaces (PVS), thus supporting the hypothesis that circulating monocytes and neutrophils can strongly contribute to the widespread dissemination of yeast cells into the CNS via a phagocyte-dependent mechanism (69). Therefore, the perivascular spaces may represent another possible entry way of *C. neoformans;* in particular, mice infected with this pathogen showed an increase in number of monocytes and neutrophils as well as in the number and size of cryptococcomas, with subsequent enhanced destruction of brain tissues; the proteolytic enzymes released by activated neutrophils contribute to damage the BBB, and this further facilitates neutrophil entry into PVS through a process of non-lytic exocytosis, allowing the yeast cells to disseminate within the CNS (56). In a subsequent stage of the infection, cerebral oedema and the enlargement of the PVS causes florid meningitis and cryptococcosis in the subarachnoid space (69); in contrast, the mice infected with *C. gattii* showed a different immunological host response with mild changes. This supports the vision according to which C. gattii has less marked neurotropism compared to *C. neoformans.*

Natural killer (NK) lymphocytes play also an important role in the innate immune defenses, as perforin secretion by these cells has demonstrated to enhance antifungal activity against Cryptococcus. Furthermore, the cytotoxicity against *C. neoformans* is increased by antibodies binding to the NK cell activating receptor CD16 (70).

Altogether, cerebral cryptococcal infection causes little or no necrosis or brain damage until later disease and, consequently, neurocryptococcosis usually presents as a subacute meningoencephalitis (71). The adult patients with neurocryptococcosis typically present neurological symptoms, including headache, altered mental status, lethargy along with fever, nausea and vomiting (71). However, in older people, these symptoms can be considered unspecific or could not to be accurately reported by patients and therefore they can be easily misdiagnosed.

## 4 Immunosenescence in the innate immune cells

The effectiveness of immune system against infections is challenging in the elderly. Aging induces significant changes in both innate and adaptive immune defense mechanisms, a phenomenon that has been defined as immunosenescence.

**Figure 2:**
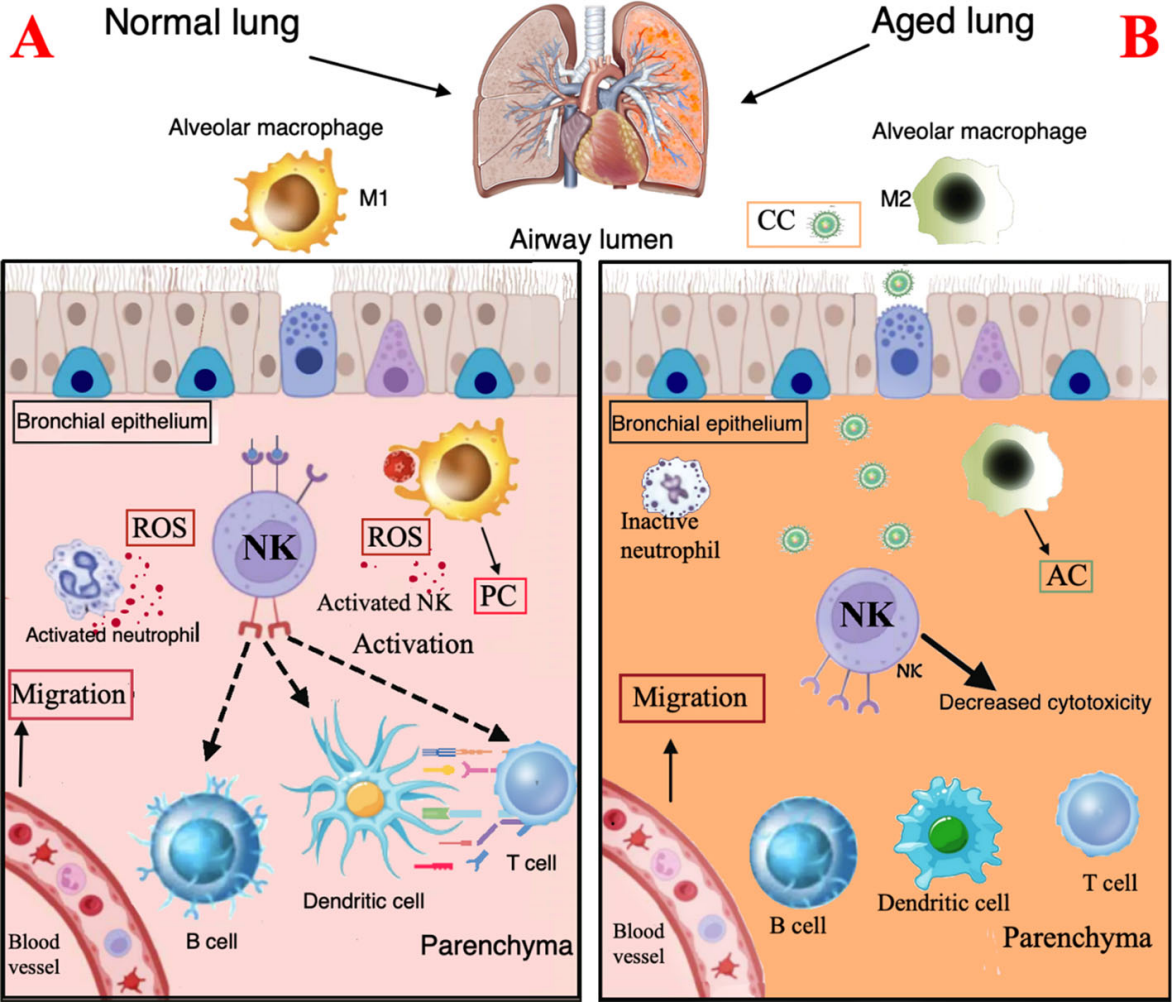
Immunological characteristics of the normal and the aging lung. **(A)**. Normal lung. Surface barriers: their integrity contributes to protect against inhaled CC. Neutrophils: activated neutrophils produce ROS and contribute to recruitment and activation of macrophages and dendritic cells. Macrophages: M1 macrophages show a proinflammatory profile and express chemotactic activity and phagocytosis ability with free radical production. **(B)**. Aging lung. Surface barriers: the decreased cilia motility is associated to higher risk of CC infection. Neutrophils, DC, and NK cells: reduced chemotactic activity, phagocytosis and ROS production. Macrophages: accumulation of M2 macrophages with anti-inflammatory profile. PC, proinflammatory cytokines; AC, anti-inflammatory cytokines; CC, Cryptococcal cells.

One of the first defense elements of the innate immune system is the lung surface barrier, which protects against inhaled bacteria, viruses, and fungi. The cilia motility decreases with aging leading to impaired mucociliary transit that may increase the likelihood of infection in the elderly (72, 73). Aging processes may also impair several functions of the innate immune cells, including neutrophils, macrophages, dendritic cells, and NK cells (74–76) (**Figure 2** **).**

*Neutrophils* are the first responders when an infection occurs. Several studies suggest that aging reduces their chemotactic activity, phagocytosis and ROS production. Moreover, TLR signal, that activates macroautophagy, appears altered; aging itself also affects the neutrophil-mediated recruitment and activation of macrophages and dendritic cells (77).

*Macrophages* orchestrate the innate and adaptive responses, thanks to their role in antigen presentation. Macrophages may have two different polarization statuses: M1 phenotype, induced by lipopolysaccharide (LPS) or interferon (IFN)-γ stimulation, shows a proinflammatory profile; the M2 phenotype, induced by IL-4, has an anti-inflammatory profile. Typically aging impairs the M1/M2 balance (78, 79); in older mice, indeed, macrophages present in hepatic and adipose tissues, have shown predominantly polarization to M1 phenotype, while accumulation of M2 macrophages occurs in lung, spleen, muscle, bone marrow, and lymphoid tissues (79–83). Age-related changes in macrophages included decreased phagocytosis, autophagy, free radical production and impaired clearance of apoptotic cells. All these modifications cause a decreased ability of macrophages to fight pathogens in older people. The reduced antigen presentation cell activity is probably due to a decreased expression of major histocompatibility complex (MHC) class II molecules, which play a key role in the initiation of the adaptive immune defense of CD4 T cells; furthermore, MHC class II expression driven by IFN-γ is impaired in macrophages of aged mice (84).

*Dendritic cells*, another type of antigen presenting cells, are responsible for the activation of NK cells and proliferation of CD4 and CD8 T cells and produce type I and III interferon (IFN-I/III). Dendritic cell functions are reduced in older adults with decreased migration capacity and impaired ability to activate the adaptive response (85). Dendritic cells and macrophages show a low TLR expression, essential for the initiation of the inflammatory signaling cascade.

Finally, *NK cell* are innate immune effectors that mediate a rapid cytotoxicity through perforin exocytosis and the production of chemokines; perforin disrupts fungal membrane integrity, aiding entry of granzymes into the fungal cell, leading to apoptosis. NK cells are key regulators of other immune cells due to their presenting antigen function, which may trigger cascade of immune responses, involving T-cells, B-cells, and dendritic cells (86). Furthermore, NK cells play a role in the recognition and elimination of senescent cells. NK cells are frequently decreased in older individuals, thus contributing to impaired microbial defense and increased risk of infection (87).

In this context, the degree of immunosencesce is also influenced by the total previous immunological changes that occurred during the lifespan and involving both innate and adaptive immunity. Indeed, the concept that memory is not an exclusive attribute of adaptive immunity is now gaining ground, as it has been observed that innate immune cells also have a sort of memory, which has been termed ‘trained immunity’. The innovative concept of immunobiography proposed by Franceschi et al. suggests that the immune system, due to its memory and plasticity, is able to register all the immunological experiences and stimuli to which the organism is exposed, which induces a continuous immune adaptation (88). In summary, all the changes in the innate immune responses of older individuals, combined with senescence of lymphocytes, lead to a decrease in resilience to address infections; moreover, impaired immune cells at BBB level combined with an age-related subtle alteration of BBB place the older patient at an increased risk of infection in the CNS. Therefore, in the next section, we will also discuss BBB changes due to aging. The alteration of BBB integrity, together with senescence of the immune system, is indeed recognized as one of the most relevant mechanisms involved in the susceptibility to neurocryptococcosis in older people.

## 5 Aging and the blood brain barrier

The BBB is a highly selective interface that regulates communication between circulation and the brain. The BBB aims to maintain CNS homeostasis, provides nourishment, and protects from unregulated exposure to blood and its contents. The BBB acts as a selective barrier that restricts the flow of many substances into and out of the CNS, also preventing unwanted toxins and pathogens from invading the brain. Proper permeability of the BBB contributes to the maintenance of functions and brain health. The BBB is subject to changes due to natural aging processes, that affect the integrity of the barrier even in the absence of underlying pathological conditions. In fact, the vulnerability of the CNS to infectious agents increases with aging, as the stability of the BBB declines and the permeability increases, thus enhancing to an increased flow of solutes, lymphocytes, and innate immune cells (89).

**Figure 3:**
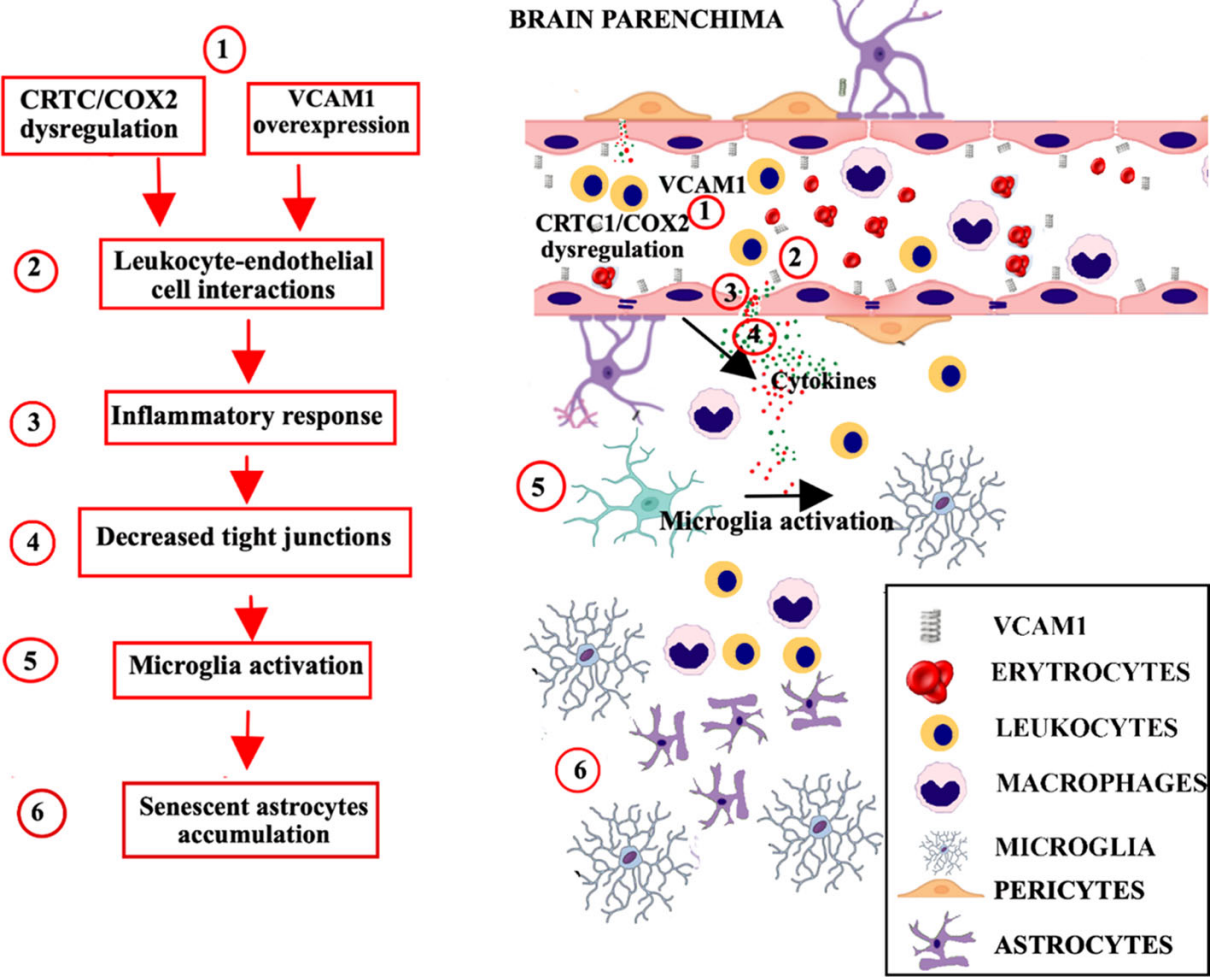
Age-related neurovascular changes in the brain parenchima and BBB. Progressive aging-related neurovascular changes in the BBB include mechanisms of inflammation mediated by the complement pathway (C3a/C3aR), with alterations to various components of the BBB structure as tight junctions, transporters, microglia, astrocytes and pericytes. **(1)**. An increase in C3a/C3aR signaling promotes the over expression of VCAM1, and a dysregulation of CRTC1/cyclooxygenase-2. **(2)**. The resulting inflammation promotes the recruitment of leukocytes across the BBB, and their interaction with endothelial cells. **(3)**. The leukocyte-endothelial cell interaction increases the BBB permeability and induces the production of proinflammatory mediators, such as reactive oxygen species and cytokines. **(4)**. Proinflammatory response leads to decreased tight junction proteins expression and conducts to peripheral immune cell infiltration. **(5)**. Proinflammatory cytokines spread outside the vessels, induce the recruitment and activation of glial cells. **(6)**. The accumulation of senescent astrocytes contributes to microglia activation, neuroinflammation and secretion of SASP factors, which communicate cellular damage to neighboring cells via autocrine/paracrine pathway. BBB, blood brain barrier; VCAM1, vascular cell adhesion molecule-1; CRTC1, CREB-regulated transcription coactivator 1.

Aging involves adaptive mechanisms in the BBB with alterations to various components of the BBB structure as tight junctions, transporters, microglia, astrocytes and pericytes (**Figure 3**).

Aging-related neurovascular changes in the BBB include mechanisms of inflammation mediated by the complement pathway. An increase in C3a/C3aR signaling promotes the expression of vascular cell adhesion molecule-1 (VCAM1), which regulates the immune cell adhesion process, and conducts to peripheral immune cell infiltration and vascular inflammation (90). Age-related inflammation and vascular cells senescence involve also CRTC1/cyclooxygenase-2 dysregulation (91). The inflammatory processes promote activation of glia cells and recruitment of leukocytes across the BBB. The leukocyte-endothelial cell interactions increase BBB permeability inducing the production of pro-inflammatory mediators such as reactive oxygen species and cytokines, which determine the alterations in paracellular and transcellular transport and decreased tight junction proteins expression (92). Also senescent erythrocytes show alterations in morphology and tightness, that consequently affects transportation and exchange of gases within the BBB and the damage mediated by reactive oxygen species (93).

Overall, several BBB modifications are favored by senescence (94). Aged pericytes show biomarkers of cellular senescence, including increased senescence-associated secretory phenotype (SASP) factors and cell cycle block (95). Accumulation of senescent astrocytes contribute to dysfunction, neuroinflammation and secretion of SASP factors, which communicate cellular damage to neighboring cells via autocrine/paracrine pathway (96).

Moreover, during aging the number of astrocytes expressing neuroinflammatory genes and the number of brain endothelial cells with high levels of senescence-related gene expression increase. Among genetic factors, the impact of ϵ4 isoform of apolipoprotein E has been associated with changes in tight-junction regulation and altered transport systems. Some BBB transporters including large neutral amino acid transporter (LAT-1), p-glycoprotein and transporters for IL-1, choline, triiodothyronine, tumor necrosis factor-alpha, glucose, Tyr-MIF-1 and enkephalins, decrease with healthy aging (97).

Furthermore, as age advances, gut microbiota dysbiosis may promote neuroinflammation causing the alteration of the brain physiopathology and BBB permeability (98). All these mechanisms may lead to BBB dysfunction and make older patients more vulnerable to neurocryptococcosis, facilitating the pathogen multiple mechanisms of barrier interaction, disruption, and CNS invasion. In this context, Cryptococcus itself is able to amplify BBB damage. Cryptococcal-mediated mechanisms of BBB permeability alteration that facilitate infection may involve the production by the fungus of urease (99), serine protease (100), metalloprotease (101), microvesicles (102), and induction of changes in the cytoskeleton of microvascular endothelial cells (103).

## 6 The putative link between immunosenescence and neurocryptococcosis: a comprehensive overview of current evidence

Although clinical evidence has pointed out the higher prevalence of neurocryptococcosis in older compared to younger adults, the exact mechanisms underlying this epidemiological evidence still need to be elucidated in depth.

However, it is very plausible that age-related disruption of innate and acquired immune response may increase the risk of both cryptococcaemia and neurocryptoccocis and may enhance the direct deleterious effects of cryptococcal infection. More in detail, aging-induced defective clearing activity of alveolar macrophages, along with increased polarization into pro-inflammatory M1 cells and increased activation of Th2 responses, are thought to contribute to increased cryptococcal damage (104, 105). Indeed, the high virulence of cryptococci is related to their capacity of escaping phagosome internalization or alternatively surviving within vesicles and strongly reactivating when immune defenses decrease (106, 107).

Overall, age-associated alterations in innate immune responses may increase the risk of ineffective cryptococcal clearance, severe pulmonary disease and dissemination to the CNS. Furthermore, aging is associated with increased expression of macrophage receptors with collagenous structure (MARCO), which are essentials in pathogen uptake by mononuclear phagocytes during early infection (108) but may be further exploited by the pathogen and polarize towards non-effective immune responses (109).

Aging also increases production of Th2 cells (110) thus creating a favorable environment for the survival of *criptococci*, mediated by the production of IL-4, IL-13, and IL-5 (105).

Furthermore, also age-related alteration of neutrophils could impact on the cryptococcal virulence. Lung neutrophils are indeed involved in degradation of pathogens as well as in the initiation of inflammation and granuloma formation against them (111), and along with dendritic cells, neutrophils also display age-driven impairment, which is more qualitative than quantitative. In fact, their number may be substantially maintained in healthy older individuals and their immune recruitment into the lungs is elevated, probably because of inflammaging (112); in contrast, the accuracy of neutrophil response towards pathogens is impaired (113–115), because of decreased specificity against the infectious stimuli, which may contribute to increased morbidity and mortality of cryptococcal infections in older hosts. Additionally, as individuals age, neutrophils produce more elastase enzymes (115), that can damage lung cells and vessels, impair macrophage immune responses (116), and potentially favoring the deleterious effects of lung infections from opportunistic microorganisms. Furthermore, decreased expression of TLR1 in aged neutrophils decrease production of IL-8 and recovery from apoptosis (117).

Overall, multiple mechanisms are involved in age-related derangement of innate immunity. In this context, several findings support the link between the higher susceptibility to infections and the increased mortality of elderly people to the emerging concept of trained innate immunity.

This theory suggests that innate immune cells possess non-specific immunological memory, whose main function is to resolve aggressions rapidly and stop the inflammatory process (118). However, after each activation, the innate immune system remains at a higher activation/readiness state (119). In older people this process is more evident, as shown by the chronic activated state of many tissue-resident and blood-born innate immune cells, including neutrophils and microglia, which display hyperactivation phenotypes in response to inflammatory stimulation, with an aberrant proinflammatory status (4, 119–121). Therefore, trained innate immunity may become hyperactivated with aging, contributing to a pro-inflammatory cytokine releasing and inflammaging. However, innate immune cells such as macrophages may also experience a decreased reactivity to different stimuli; thus, trained immunity may undergo a decline, contributing to immunosenescence. Factors that lead trained innate immunity to a weakened or hyperactivated state have not been clearly identified. However, the concept of immunobiography, which is based on the development of an immunological history, could explain the heterogeneity of responses in the elderly toward one form rather than the other (88).

The hyperactivation of inflammatory signaling pathways, associated to a dysregulation of proinflammatory mediators and defined as inflammaging, might lead to a relative immune paralysis state, characterized by markedly impaired innate immune function against pathogens (120, 122, 123).

In this framework, dysregulation of IFN-I response may represent a phenotype of late senescence and appears to contribute to the maintenance of the age-associated inflammation (124). Indeed, on one hand, aging and age-related comorbidities may impair IFN-I production thus leading to ineffective viral clearance and increased susceptibility to infections during the first contacts with the pathogens (5); on the other hand, paradoxically, various aging tissues and organs from mammalian hosts perpetually accumulate changes brought by IFN-I pathway activation (20). Therefore, IFN-I may contribute to the increased neuroinflammatory status characterizing aging of the brain (125–127), which is frequently enhanced by the presence of comorbidities (128, 129). Accordingly, IFN-I signaling has been shown to be up-activated in the choroid plexus in the aged CNS of humans and mice (130, 131).

In summary, during aging we may recognize a progressive establishment of an “interferonopathic disorder”, that is an IFN-mediated inflammatory status mainly characterized by a severe age-related neuroinflammation and IFN-I up-regulation, a phenomenon also known as IFN-aging (20, 132). In addition to aging-related impairment in IFN-I responses, many comorbidities associated with aging are known to contribute to elevated IFN-I levels. However, so far studies concerning the role of IFN-Is during fungal infections have generated conflicting results. A host protective contribution of IFN-Is to immunity against *C. neoformans* was highlighted by some studies showing that IFNα/β deleted mice were more susceptible to infection than wild-type mice (133–135). On the other hand, some investigations reported that the clearance of *C*. *neoformans* in the mouse lungs was accelerated at the early phase of infection, under a condition lacking IFN-I-mediated signaling, suggesting that IFN-Is may be involved in negative regulation of the early host defense (136). Probably, the role of IFN changes in the context of cryptococcal infection from a protective to a deleterious role depending on the phase of the infection, the virulence of the pathogen and the general performance status of immune system.

**Figure 4:**
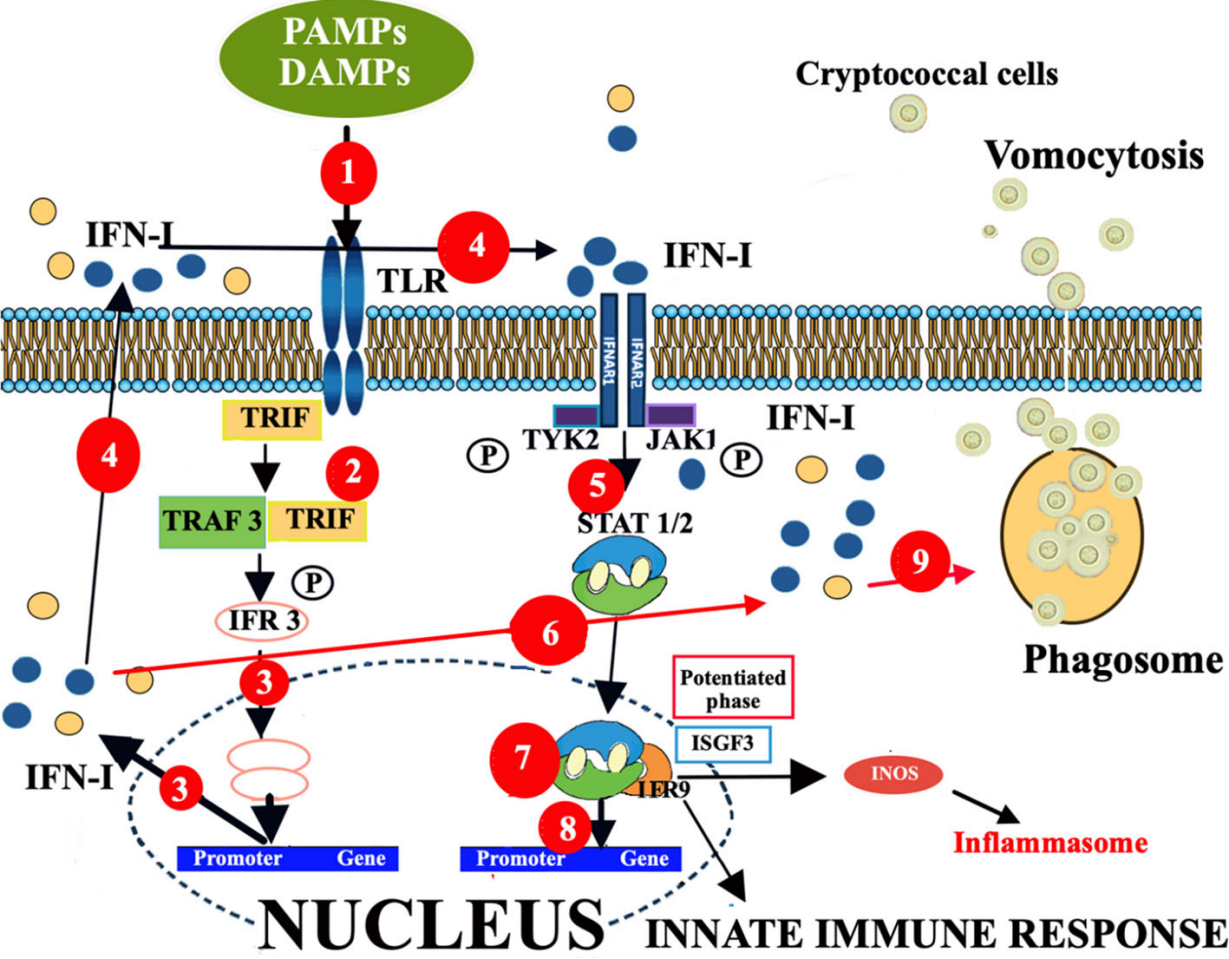
Enhancement of vomocytosis via IFN-I mediated pathway. The different effects of IFN-I signaling, depending on ligand, dose, and duration of exposure. **(1)**. The TLR-PAMP/DAMP interactions induce the selective recruitment of TRIF, which binds to the TLRs and then recruits a series of downstream signaling molecules, leading to IFN-I production. **(2)**. TLR-PAMP/DAMP interactions induce TRIF to form a signaling complex with TRAF and this leads to phosphorylation of IRF3. **(3)**. This phosphorylation event causes IRF3 to dimerize, translocating into the nucleus, and inducing the expression of IFNα and IFNβ. **(4)**. Both secreted IFN-Is can bind IFNAR1/2 in an autocrine or paracrine manner and activate a signaling cascade leading to expression of ISGs. **(5)**. In particular, the c-termini of IFNAR1 and IFNAR2 are associated with TYK2 and JAK1, respectively, and activation of the receptor transduces the phosphorylation of JAK1 and TYK2 by tyrosine phosphorylation. **(6)**. This initiates a signaling cascade composed of proteins of the STAT family. **(7)**. STAT1 and STAT2 proteins, activated upon JAK1 phosphorylation, dimerize and rapidly translocate to the nucleus, where they, together with IRF9, form a trimolecular complex called IFN-stimulated gene factor 3 (ISGF3). ISGF3 is a critical transcription factor complex involved in the cellular response to IFN-Is, particularly IFN-α and IFN-β. **(8)**. This complex activates the transcription of ISGs. **(9)**. However, IFN-I responses may also induce innate immune cells to act as “vomocytes”, giving rise to an ineffective fungal clearance which leads to a latent asymptomatic lung infection. In any case, this ISGF3 engagement by IFN-I production leads to activation of the inflammatory gene expression program, responsible for activation and/or exacerbation of inflammatory responses. Altogether, ISGs may produce, via JAK-STAT/IRF signaling network, distinct biological responses and sustained IFN-I signaling leads to chronic immune activation, inflammation and, consequently, immune exhaustion and dysfunction. Consequently, during infections, the effects of IFN-I signaling can be protective or detrimental, depending on the context, including pathogen species, infection route, and tissue specific features. DAMP, damage-associated molecular patterns; IFNAR1/2, IFN-α/β receptor; IFN-I, interferon; IRF, interferon regulatory factor; ISGs, IFN-stimulated genes; ISGF3, interferon stimulated gene factor 3; ISGF9, interferon stimulated gene factor 9; JAK1, Janus kinase; PAMP, pathogen-associated molecular patterns; STAT, signal transducer and activator of transcription; TRAF, TNF receptor-associated factor; TRIF, Toll/IL-1R domain-containing adaptor-inducing IFN-β; TYK2, tyrosine kinase 2.

To this regard, it is important to take in mind that key features of cryptococcal pathogenesis are the ability of the fungus to survive and replicate within macrophages; then they are typically expelled by a mechanism of non-lytic exocytosis called vomocytosis and/or by cell lysis (64). Vomocytosis has been shown to be enhanced by IFNα and/or IFNβ, and abrogated when IFN-I signaling is blocked (137). Essential features of IFN-I signaling are shown in **Figure 4**.

In brief, the PRR-PAMP/DAMP interactions induce the selective recruitment of the Toll/IL-1R domain-containing adaptor-inducing IFN-β (TRIF), which binds to the TLRs and then recruits downstream signaling molecules that ultimately induce the production of IFN-I (138). In particular, TRIF forms a signaling complex with TNF receptor-associated factor (TRAF) and this leads to phosphorylation of the transcription factor interferon regulatory factor (IRF)3 (139). This phosphorylation event causes IRF3 to dimerize, translocate into the nucleus, and induce the expression of IFNα and IFNβ. Both secreted IFN-Is can bind the IFN-α/β receptor (IFNAR1/2) in an autocrine or paracrine manner and activate a signaling cascade leading to expression of IFN-stimulated genes (ISGs), via JAK-STAT1/2 signaling (**Figure 1**).

It is hypothesized that in a first phase of cryptococcal infection, IFN-I responses may induce innate immune cells to act as “vomocytes”, giving rise to an ineffective fungal clearance which leads to a latent asymptomatic lung infection that may be considered as a host tolerance mechanism able to prevent or limit the tissue damage and the systemic dissemination (135). Consequently, whereas cryptococcal vomocytosis at an early stage of infection might be beneficial, since it would limit the risk of systemic fungal shedding, once the fungus has reached the blood stream or CNS, enhanced vomocytosis is likely to be deleterious, since it would allow the pathogen to escape the constraints of a phagocyte and grow rapidly in the extracellular environment.

In addition to the putative dysregulation of IFN-dependent molecular pathway, other molecular mechanisms are probably involved in the increased susceptibility to neurocrytococcosis in the older people compared to young individuals. For instance, an invertebrate model of *C. neoformans* and *C. gattii* infection suggested that defects in both longevity and innate immunity, affecting the insulin/IGF-1 signaling/DAF-16 pathway, has been linked to a raised susceptibility to Cryptococcus (140). Furthermore, the authors reported that *C. neoformans or C. gattii* infections reduced the host lifespan. Another study in rats showed that lung macrophages, after *C. neoformans* phagocytosing, induce the synthesis of monocyte chemotactic protein 1 (MCP-1), depending on the interaction of *C. neoformans* with CD11b/c and CD18 and, interestingly, aging appears to be related to a reduced production of MCP-1 by lung macrophages in response to cryptococcal infection (141). Another evidence suggesting this link between immunosenescence and cryptococcal infection is the increased risk for cryptococcosis associated with long-term use of fingolimod (142). Indeed, this drug induces phenotypic changes on many cells of the immune system similar to the immunosenescence observed in the older people.

In addition to systemic immunological alterations, also at CNS level, in the context of cryptococcal infection, an aberrant immune response probably contributes to the damage. To this regard, a small cohort of 17 middle-age HIV-negative adults with severe CNS cryptococcosis have shown an intrathecal expansion and activation of dendritic cells and lymphocytes (CD4+, CD8+ and NK cells) and high levels of inflammatory cytokines including IFN-γ and IL-6, leading to the idea that the increased inflammatory response in neurocryptoccocosis doesn’t represent a protective defense, but enhances markers of neuronal injury (143). These results appear to be similar to the increased immune reconstitution inflammatory syndrome (IRIS) in HIV-infected people or post-infectious inflammatory response syndrome (PIIRS) in HIV-negative people occurring occasionally with cryptococcal meningitis. A case of PIIRS was also recently described in an elderly patient, who showed increased IL-6 levels in the serial cerebrospinal fluid (CSF) (144).

Finally, immunosenescence has shown to affect also the adaptive immune response to fungal infections. In this regard, age-related T-cell dysfunctions in elderly mice have been reported to be related to an increased susceptibility to systemic cryptococcal infection compared with young adult mice (145).

Altogether, these findings underscore the importance of studying human immune responses at different stages of infection and in different immunological conditions.

## 7 Future directions in clinical research: diagnosis and therapy

The significant association between aging and neurocryptococcosis implies a need to adopt appropriate prevention measures.

Older populations may be more vulnerable to diagnostic error for several reasons, including an incorrect attribution of symptoms to normal aging, or to unrelated neurological findings.

Moreover, accurate diagnosis is also critical in older people, who, in addition to concomitant neurological disorders, have a slower onset of symptoms, evolving over days to a few weeks, condition that could make it difficult an early diagnosis and lead to a delay in treatment (15). Furthermore, the clinicians sometimes could focus unduly on clinical clues suggesting specific diseases, while discounting opposing clues, and sometimes they could confound meningitis with other disorders sharing similar symptoms, including achy stiffness, headache, and high fever. Misdiagnosis not only endangers the safety and health of older patients, but also incurs additional costs for health care systems due to misused clinical interventions and care of iatrogenic illness.

The clinical suspicion of neurocryptococcosis should be followed by targeted diagnostic investigations, including first-line computed tomography and magnetic resonance imaging; subsequently, lumbar puncture for CSF analysis should be performed to confirm the diagnosis; a positive CSF culture indicates active cryptococcal disease and represents the gold standard method to detect live pathogens; additional CSF analyses, including India ink preparation and Gram staining, can help quantify the degree of capsule thickness and melanization of the yeast cells cultures, which are related to fungal virulence and immunodepression of the host (146). In contexts where lumbar puncture cannot be rapidly performed, clinicians can rely on the positivity of the cryptococcal antigen in the serum, which can strongly suggest the infection, even if the diagnostic gold standard is the CSF culture. However, it is important to take in mind that, when the fungal burden is low, CSF fungal culture can produce false negative results, as well as India ink microscopy. Furthermore, the slow-growing nature of Cryptococcus as well its possible physiological viable-but-nonculturable (VBNC) status (147) may contribute to false negative CSF cultures. In any case, despite obtaining a reliable result may require several weeks, CSF fungal culture remains paramount for the definitive diagnosis of neurocryptococcosis.

The therapeutic approach also requires careful evaluation, since antifungal drugs are particularly toxic and could aggravate the basic medical conditions of frail older subjects suffering from multiple comorbidities. The principal antifungal agents classically used for the treatment of neurocryptococcosis consist of intravenous amphotericin B deoxycholate and its lipid formulations, oral flucytosine, and oral fluconazole (28). While amphotericin B and flucytosine are fungicidal, fluconazole is only fungistatic. Lipid formulations of amphotericin B are preferred on older patients often affected by renal dysfunction or at risk for renal failure. The high prevalence of multimorbidity and polypharmacy among older adults, that contributes to the relatively increased risk, associated to both the polypharmacy and the frail conditions, implies a need for a very careful evaluation also concerning the risk/benefit ratio in the different frail conditions of affected patients.

In particular, it should be taken in consideration that, older patients using antimicrobials have a higher risk of adverse side effects due to age-related changes in pharmacokinetic and pharmacodynamic, multimorbidity, and polypharmacy (148). More specifically, the use of amphotericin B is typically associated with a higher risk of nephrotoxicity, hepatotoxicity, hematological effects, and heart failure. Nephrotoxicity is the most common potential adverse effect in older patients, presenting with an increased creatinine level, hypo/hyperkalemia, and/or hypomagnesemia (148). In addition, although the lipid formulations have been shown to be substantially less toxic than conventional amphotericin B, particularly with respect to nephrotoxicity, concomitant or recent use of other nephrotoxic drugs could have an additive effect (148).

With respect to flucytosine, despite leukopenia and myelosuppression represent side effects frequently induced by high plasma levels of this drug, maintained for a long time, the current use of flucytosine is allowed for the treatment of cryptococcal meningitis, in combination with amphotericin. However, the concurrent use of flucytosine and nephrotoxic drugs should be avoided due to the risk of accumulation of flucytosine (149). Furthermore, clinicians should be aware that fluconazole, even at low doses, may cause cardiotoxicity for prolongation of the QT interval (148).

In addition to currently available anti-fungal drugs, it would be desirable for new therapeutic strategies to be available in the next future for neurocryptocococosis. To this regard, some investigations, based on pre-clinical findings, have tested commercially available drugs used for other indications, including tamoxifen and sertraline as potential treatment for cryptococcal infection, according to the so-called repurposing strategy (150, 151). However, until now these studies did not show any efficacy of these treatments against cryptococcal infection.

In our opinion, considering the putative role of immunosenesce in the increased susceptibility of older people for neurocryptococcosis, potential therapeutic approach could be based on the regulation of immune response. For instance, some strategies aimed to modulate CD22 receptor on microglia seem to be able to restore homeostatic microglial phagocytosis in ageing brain (152).

Another putative target to contrast immunosenescence is inhibition of p-38, that is a molecule strongly implicated in the aberrant inflammation due to age-related macrophagic dysfunction (153, 154).

However, all these approaches need to be tested in the context of cryptococcal infection.

## 8 Conclusions

Given the high complexity of neurocryptococcosis in older individuals, we suggest that a multidisciplinary and comprehensive approach to the disease to be followed; cryptococcosis presents unique challenges in older individuals due to age-related changes in immune function and increased susceptibility to infections. As our population continues to age, it is imperative that healthcare professionals remain vigilant in recognizing and managing cryptococcal infections in this vulnerable population. Early diagnosis, prompt treatment, and close monitoring are crucial for improving outcomes and reducing morbidity and mortality associated with cryptococcosis in older adults. Additionally, ongoing research is needed to better understand the epidemiology, pathogenesis, and optimal management strategies tailored specifically for older individuals.
